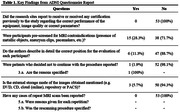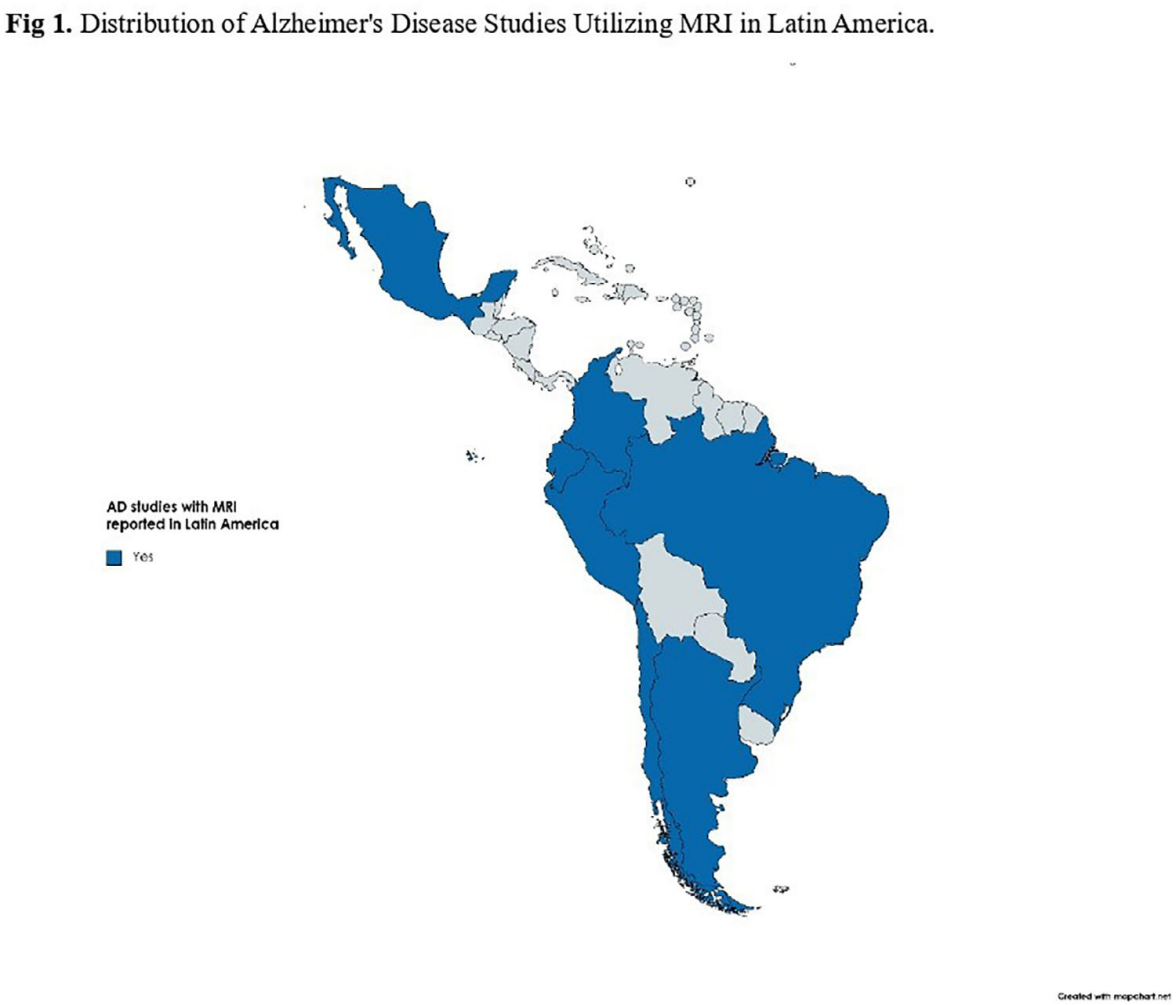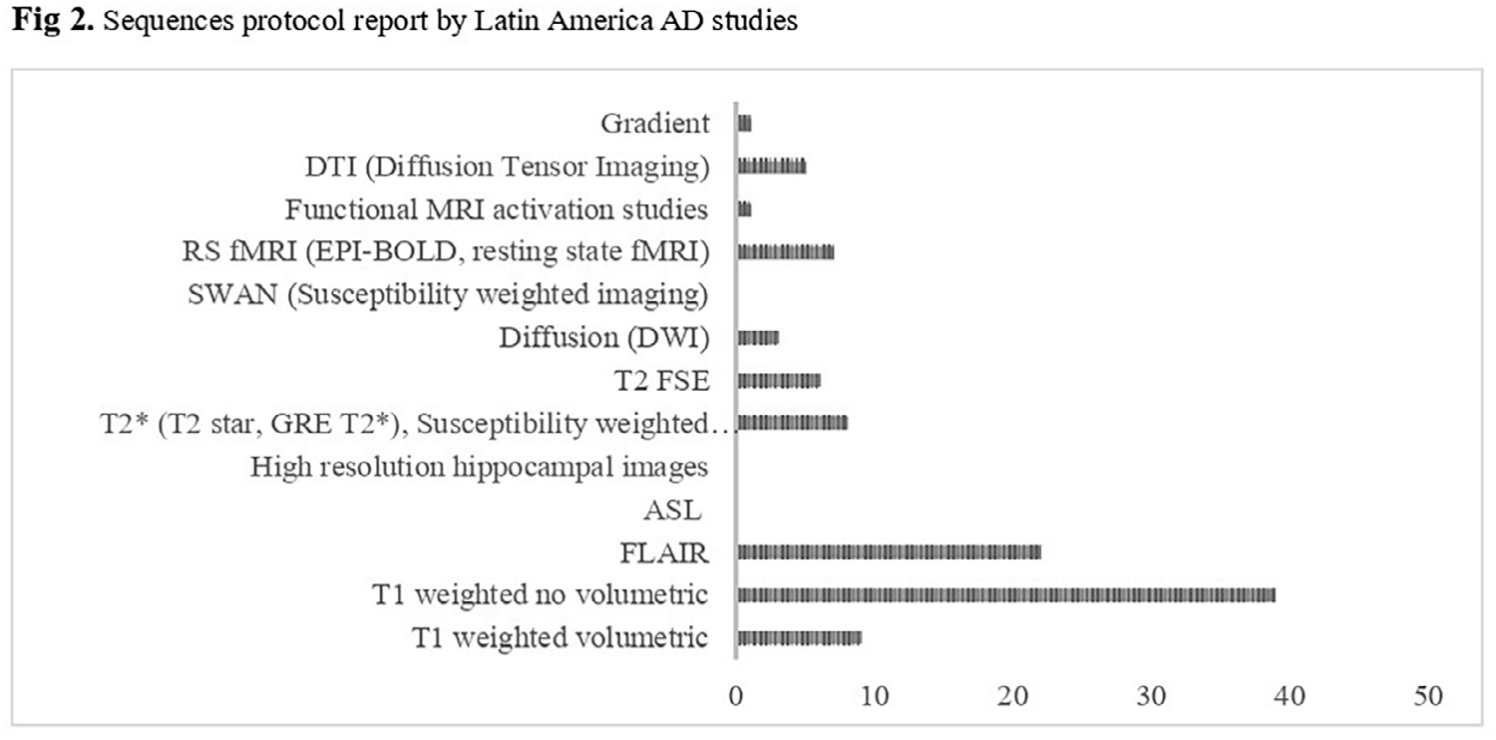# Use of MRI for Alzheimer’s Disease Across Latin American Countries: A Systematic Review

**DOI:** 10.1002/alz.091926

**Published:** 2025-01-09

**Authors:** Marco Malaga, Valeria Rivera, Valeria Morales, Diego Bustamante‐Paytan, Andres Damian, Margarita García‐Fontes, Rosa Montesinos, Nilton Custodio

**Affiliations:** ^1^ Cognitive Impairment Diagnosis and Dementia Prevention Unit, Instituto Peruano de Neurociencias, Lima, Perú, Lima, Lima Peru; ^2^ Grupo de Investigación Neurociencia Efectividad Clínica y Salud Pública, Universidad Científica del Sur, Lima, Lima Peru; ^3^ Grupo Estudiantil de Investigación en Neurociencias, Sociedad Científica de Estudiantes de Medicina de la Universidad de San Martin de Porres, Lima, Lima Peru; ^4^ Universidad de San Martín de Porres, Lima, Lima Peru; ^5^ Cognitive Impairment Diagnosis and Dementia Prevention Unit, Peruvian Institute of Neurosciences, Lima, Lima Peru; ^6^ Centro Uruguayo de Imagenología Molecular, CUDIM., Montevideo, Montevideo Uruguay

## Abstract

**Background:**

The worldwide prevalence of Alzheimer's Disease (AD) surpasses 55 million, with Latin America reaching 11%, driven by demographic changes. Precise diagnosis necessitates neuroimaging, particularly magnetic resonance imaging (MRI). However, low‐ to middle‐income countries face challenges in MRI accessibility due to limited imaging equipment and a shortage of specialized healthcare professionals trained in neuroimaging.

**Methods:**

A systematic literature in English and Spanish was conducted in three databases for AD studies where MRI was conducted. Fifty‐three studies were included based in the inclusion and exclusion criteria. A narrative summary of protocols sequencing, and qualitative analysis was performed. For quality assessment, we evaluated the reporting of their imaging methodology based in the ADNI 3 study imaging protocol version 1.

**Result:**

A total of 2370 reports were initially identified. Fifty‐three studies were ultimately included in the review, predominantly originating from Brazil (Figure 1). Within these studies, 38.6% employed the TI‐weighted non‐volumetric sequence (Figure 2). Functional analysis, regional brain volume, and white matter lesion quantification were reported in 20%, 20%, and 22% of the studies, respectively. Notably, research sites omitted information on whether they had obtained proper certification for health professionals and imaging equipment before the study. Most studies (71.7%) did not disclose whether pre‐scan contraindications evaluations were conducted, and only 11.3% specified the correct position for each participant during MRI subject pre‐scan procedures. In terms of external storage mode for acquired images (e.g., DVD, CD, online cloud, etc.), only three studies (5.7%) provided this information. As for scan procedures, merely one study (1.9%) documented instances where patients chose not to continue with the procedure, and no studies reported cases of repeat MRI scans (Table 1).

**Conclusion:**

Significant and concerning gaps exist in Latin America regarding the acquisition of images for diagnosing AD. The development of standardized protocols is imperative to enhance the quality of neuroimaging outcomes in Alzheimer's Disease studies within this region.